# Rehabilitation of Worn Dentition with CAD-CAM Restorations: A Case Report

**DOI:** 10.3290/j.jad.b2916447

**Published:** 2022-04-13

**Authors:** Cees M. Kreulen, Luuk A.M.J. Crins, Niek J.M. Opdam, Bas A.C. Loomans

**Affiliations:** a Assistant Professor, Radboud University Medical Center, Radboud Institute for Health Sciences, Department of Dentistry, Nijmegen, The Netherlands. Protocol development, clinical case operator, wrote the manuscript.; b Lecturer and Dentist, Radboud University Medical Center, Radboud Institute for Health Sciences, Department of Dentistry, Nijmegen, The Netherlands. Validation, investigation, project administration, wrote the manuscript.; c Professor, Radboud University Medical Center, Radboud Institute for Health Sciences, Department of Dentistry, Nijmegen, The Netherlands. Project leader Radboud Tooth Wear Project, protocol development, project administration, wrote the manuscript.

**Keywords:** CAD-CAM, case report, digital workflow, indirect composite materials, minimally invasive, oral rehabilitation, restorative dentistry, tooth wear

## Abstract

**Purpose:**

To describe the digital workflow applied for restoring a severely worn dentition with minimally invasive CAD/CAM resin nano-composite restorations.

**Materials and Methods:**

A 40-year-old male in good general health and with full-arch dentition suffered from dentin hypersensitivity and wanted to improve the esthetics of his worn anterior teeth. The dental wear can be described as general, grade 3, according to the Tooth Wear Index,^27^ with more wear in maxillary than in mandibular teeth. Signs and symptoms were typical for a chemical type of wear, with some mechanical wear also apparent. No functional problems, eg, impaired chewing, were present. On the OHIP-49 questionnaire, the patient expressed a reduced quality of life. The goal of the treatment was to reconstruct the anatomical form of the teeth as far as possible, thereby also improving quality of life. Due to the rather large volume of lost tooth tissue per tooth, indirect treatment using CAD/CAM resin nano-composite restorations (LAVA Ultimate, 3M Oral Care) was applied.

**Results:**

The seating of the CAD/CAM resin nano-composite restorations (LAVA Ultimate, 3M Oral Care) restorations was considered precise.

**Conclusion:**

In the treatment of severe tooth wear, the described digital workflow using CAD/CAM restorations for occluding restorations and direct composite materials in the esthetic zone is a potential treatment modality that is workable and minimally invasive.

Significant clinical symptoms of patients with severe generalized tooth wear include tooth sensitivity, chewing difficulties, impaired esthetics, and fracture of tooth tissue and dental restorations.^15,28^ Given these problems, the outcome of tooth wear management, including shared decision making with the patient, can lead to restorative intervention to rebuild the worn dentition to its original anatomy.^15^ Full crowns may be the restorations of choice in such reconstruction,^3,11,22^ as all restorations can be designed coherently in one technical procedure; moreover, indirect restoration techniques in dentistry have a long, successful history. However, full-mouth clinical preparation procedures are strenuous, and full-coverage crowns entail high biological and economical investments and are therefore considered as invasive treatment.^8^ Moreover, as severe tooth-wear patients can be considered at high risk of restoration failure, it can be expected that any restorative treatment is likely to have a limited longevity. While candidates for those full rehabilitations are often relatively young, this results in retreatments of increasing complexity.

To minimize the biological price, tapered tooth preparations should be avoided and, as the tooth has already been worn away, one should preserve tooth tissue in order to enable future retreatments with minimal risk of complications. Minimal-intervention rehabilitations include restorations with direct composites.^10,16-19,26^ These treatments show satisfying mid-term results, although repairs may be necessary in the follow-up, as described in recent systematic reviews.^5,13^ Moreover, rehabilitations with direct composite require technical skills of the operator and considerable clinical working time.

To reduce the demand on the operator’s skills, indirect adhesive restorations may offer another minimally invasive treatment modality to restore worn dentitions. Another advantage compared to direct techniques may be the more predictable adaptation of the vertical dimension of occlusion (VDO) in the treatment procedure. Recently, encouraging results have been reported using polymer-infiltrated ceramic networks (PICN) manufactured by CAD/CAM techniques for dental reconstruction in tooth-wear patients, including bruxists, following a minimally invasive protocol.^23^ Another recent report showed excellent results with lithium-disilicate glass-ceramic restorations, although in that study,^7^ a moderately invasive technique was still employed, including occlusal and proximal reduction of teeth.

The mechanical properties of CAD/CAM composite have been optimized, as the material is in the maximum polymerized condition. This improves wear resistance of the restorative material, while maintaining a dentin-like elastic modulus.^14^ The material is milled in the maximum polymerized condition, which promotes optimal fit of the restoration, since no postprocessing compensation is required. If combined with a digital 3D-impression made with an intraoral scanner, an almost completely digital workflow can be followed, which might be beneficial if multiple build-up restorations (eg, uplays and backings) in one patient have to be produced.^9,23^

Today, minimally invasive techniques for the treatment of severe tooth wear may not exclusively be restricted to direct restorations, since CAD/CAM processing of combined resin-ceramic materials seem to offer clinically functional materials as well as operator-friendly technical procedures. The aim of this case report was to describe the digital workflow that was employed for restoring a severely worn dentition with minimally invasive CAD/CAM resin nano-composite restorations.

## Case Report

**Fig 1:**
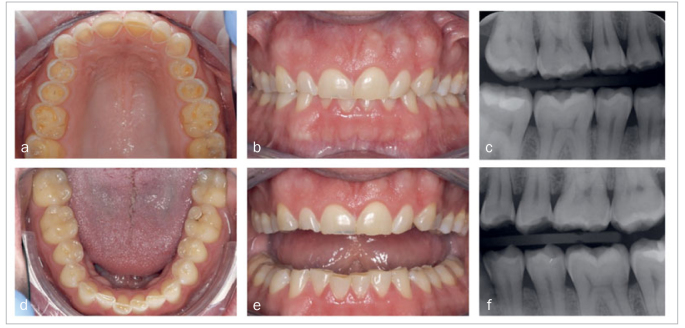
Intraoral photographs and bitewing radiographs at baseline.

The patient was a 40-year-old male with good general health and full-arch dentition who worked as a chef in a local restaurant. He was made aware of the dental wear by his dentist and was concerned about its progression. At the first appointment, anamnestic information was acquired and the level of tooth wear was documented using intra-oral photographs (Fig 1). The patient was informed about the multifactorial etiology of tooth wear in general. Parafunctional habits (grinding) and frequent acidic intake, related to his profession, were identified. The patient was not aware of signs of gastroesophageal reflux disease (GERD) at first, but acknowledged the presence of mild symptoms later. The decision was made to start restorative treatment, as the patient had a clear demand for treatment due to dentin hypersensitivity and wanted to improve the esthetics of the worn anterior teeth. To reduce progress of clinical symptoms and to optimize the long-term peformance of restorations, he was advised to limit acidic intake and to use a fluoride-rich mouthrinse daily. The patient was also referred to his general medical practitioner to limit reflux of gastric acid.

No functional problems, eg, impaired chewing, were present. There were no observable periodontal pockets. Oral hygiene was adequate, as there was hardly any visible plaque and no bleeding on probing. Risk assessment for caries was performed and judged to be low, as only a few occlusal composite restorations were present and no carious lesions were detected. He had an Angle Class I molar relation with an active occlusion that could be characterized as group function to both the left and the right side (Fig 1).

**Fig 2:**
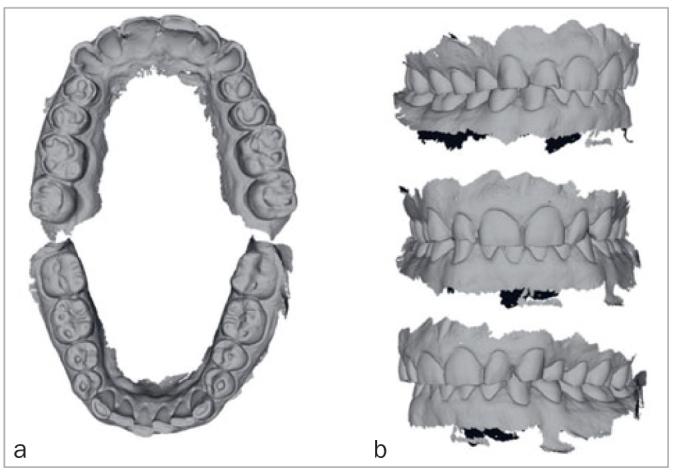
Intraoral 3D scans, occlusal surfaces (a), and maximal occlusion (b).

The dental wear was described as general, grade 3, according to the Tooth Wear Index,^27^ with more wear in the maxillary than in the mandibular teeth. Central incisors were worn away for about 2 mm of length. Based on the gingival line of the mandibular front teeth, dentoalveolar compensation was not apparent. Although mechanical wear as an etiological factor could not be ruled out, signs and symptoms were typical of chemical wear (eroded palatal surfaces, deep cupping, no wear facets on occluding surfaces, dentin hypersensitivity). This is also shown on the intraoral scans (Fig 2). On the OHIP-49 questionnaire, the patient expressed a reduced quality of life.

In this case, severe and generalized occlusal wear was present, which resulted in shortening of the teeth. An increase of VDO (vertical dimension of occlusion) by restorative rehabilitation was required. The treatment goal was to reconstruct, as much as possible, the anatomical form of the teeth, thereby also improving quality of life. Due to the considerable volume of lost dental tissue per tooth, the decision was made to use an indirect treatment technique with CAD/CAM restorations.

### Clinical Try-out Session To Determine the New VDO

**Fig 3:**
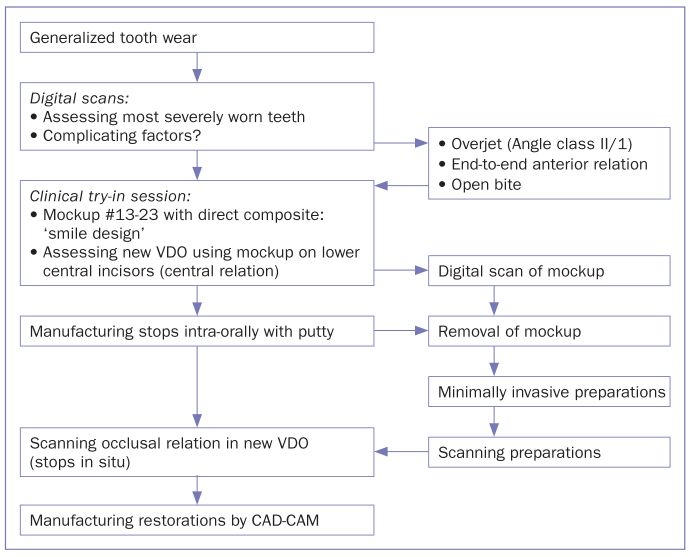
Workflow to determine the new VDO in relation to the clinical procedures.

**Fig 4:**
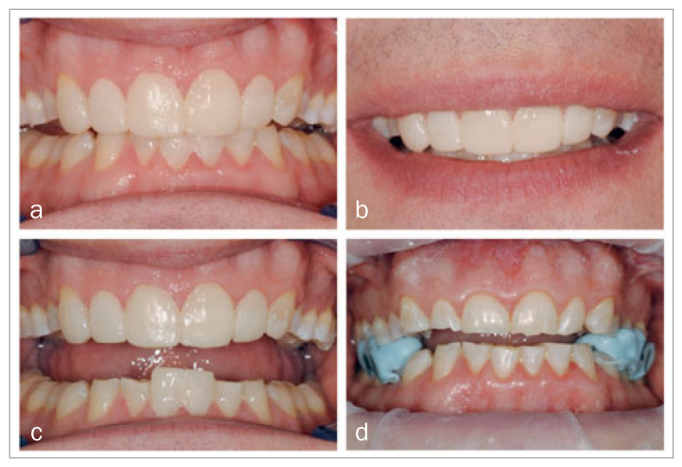
Mockups on the maxillary and mandibular anterior teeth and silicon stops in situ to preserve the new VDO.

The work flow to determine the VDO that we used is presented in Fig 3. The new VDO was estimated based on the lost anatomical shape of the teeth and the necessity to lengthen the maxillary and mandibular anterior teeth. The starting point of reconstruction was an in situ free-hand mockup using direct, unbonded composite anterior veneer restorations according to the lip-generated smile design.^20^ We used the mockup to determine whether lengthening the anterior teeth was clinically applicable and esthetically satisfactory. To provide the patient a realistic impression of the expected esthetic appearance, the mockup included all 6 maxillary anterior teeth. In this way, the location of incisal edges relative to the lip, smile, and color of the potential restorations could directly be checked by both operator and patient (Figs 4a and 4b). After approval of the esthetics by the patient, the mockup was recorded using a digital pre-op scan (True Def IOS, 3M Oral Care), serving as guidance for the digital wax-up in a later stage of the procedure.

Then, the estimated new VDO was determined intraorally by applying a free-hand composite mockup on the central mandibular incisors (Fig 4c). By applying composite to the palatal surfaces of the maxillary central incisors, we controlled the interocclusal distance at the location of the first molars when the patient was closing along the retruded path. This led to a VDO increase of about 3.5 mm (roughly 1.5 mm space for the required thickness of the maxillary and mandibular restorations). This VDO was preserved by making fast-setting, stiff silicon bite blocks (Star VPS, Danville Materials; San Ramon, CA, USA) in the right and left posterior areas while the patient was in centric relation closing on the anterior mockups (Fig 4d). After the mockups were removed, composite restorations on the occlusal surfaces of teeth number #37 and #47 were replaced with direct composite restorations due to deficient margins.

### Preparation and Scanning

**Fig 5:**
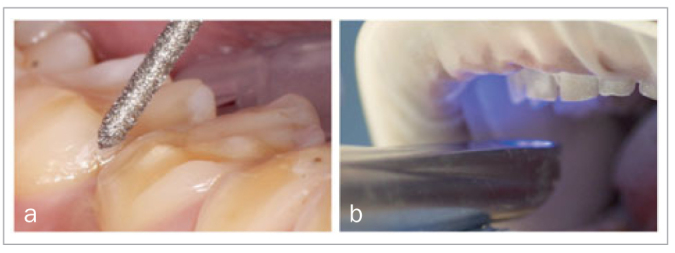
Preparation of teeth and intraoral scanning.

During the next treatment session, all teeth were prepared for indirect restorations: table-tops on posterior teeth, palatal veneers on maxillary anterior teeth and buccal veneers on mandibular anterior teeth. Tooth preparation was limited to 1) removing sharp ridges that could initiate cracks in the covering restoration, and 2) a small chamfer to provide the outline of posterior occlusal surfaces (Fig 5a).

**Fig 6:**
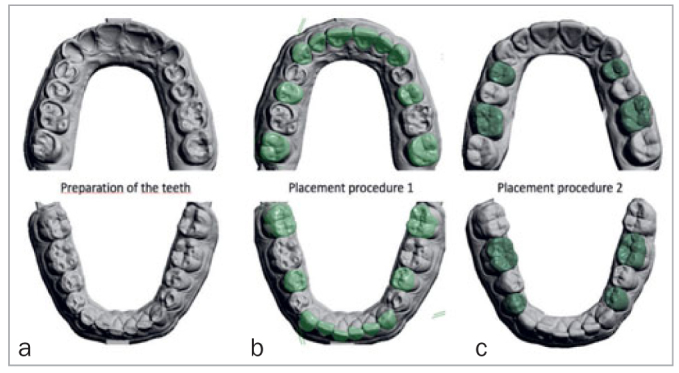
Design of the indirect composite restorations according to two procedures.

Digital impressions (True Def IOS, 3M Oral Care) were made according to the manufacturer’s instructions (Figs 5b and 6a). Restorative surfaces of maxillary anterior teeth were located just supragingivally on the palatal surfaces, so gingival retraction was necessary during scanning for these teeth only. After the maxillary and mandibular scans were obtained, the occlusion scans with increased VDO were made with the two bite blocks in situ. In this way, the new VDO was transferred from the clinic to the digital environment. Since hardly any occlusal tooth tissue was removed, temporary restorations were not necessary.

Scans were transferred to the dental laboratory (Elysee Dental, Modern Dental Laboratory; Alphen aan de Rijn, The Netherlands), which designed a fully digital wax-up. The oral and buccal surfaces of the maxillary anterior teeth were designed according to the scanned mockup (pre-op scan of mockup). At this stage, the mandibular anterior teeth, the second premolars, and the second molars, both in the maxilla and in the mandible, were designed (procedure 1, Fig 6b). It is important to note that the first premolars and first molars were designed after the placement of the restorations of the second (pre)molars. After the dentist approved the design, veneer restorations including the incisal edges for the anterior teeth and table-top restorations for the posterior teeth were CAM-milled from a resin nano-composite material (Lava Ultimate, 3M Oral Care, color A2) by the dental technician (Elysee Dental).

### Placement Procedure 1

**Fig 7:**
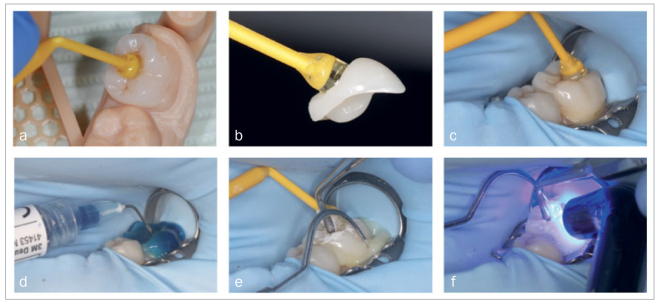
Cementation of the CAD-CAM restorations. a: indirect restoration placed on printed model; b: indirect restoration adhering to adhesive stick; c: try-in of indirect restoration; d: etching of the abutment tooth; e: placement of indirect restoration and removal of excess cement; f: light curing of cement.

For the delivery of the restorations and global check of seating, a printed model was used to fit the milled restorations. The steps before placement included cleaning of the adherent surfaces of the teeth with pumice, then rinsing. Restorations were tried-in prior to cementation with the restoration adhered to an adhesive stick (Optra stick, Ivoclar Vivadent; Schaan, Liechtenstein) (Fig 7). The internal surface of the restorations was sandblasted using SiO_2_-coated Al_2_O_3_ (Cojet, 30 µm, 3M Oral Care) under 2.5 bar to clean, roughen, and apply a silicate coating on the surface, followed by a layer of silane (ESPESIL, 3M Oral Care).

Rubber-dam was applied, and a transparent matrix was wrapped around the tooth and fixed with wooden wedges. The tooth was etched (Scotchbond Etchant, 3M Oral Care) for 15 s, rinsed, and air dried. Then the bonding agent (Scotchbond Universal, 3M Oral Care) was applied, gently air dried, and photocured for 10 s. The restorations were placed using composite cement (RelyX Ultimate, 3M Oral Care), excess cement was removed, followed by light curing for 40 s (Bluephase 16i, Ivoclar Vivadent; maximum output 1600 mW/cm^2^). Finally, the outline of the restorations was finished using fine diamond burs, finishing disks, and finishing points.

In this procedure, placement started with the maxillary central incisors, followed by the lateral incisors, canines, second premolars, and second molars. Subsequently, restorations of the mandibular anterior teeth were placed in the same sequence. After removing occlusal interferences, the remaining teeth (first premolars and first molars) were cleaned with pumice and the preparations were adjusted. New, full intraoral scans – including bite registrations – were made and sent to the dental laboratory to design and produce the eight remaining indirect restorations for procedure 2 (Fig 6c).

### Placement Procedure 2

The patient revisited the clinic about two weeks after placement procedure 1. The procedures to check the seating of the remaining eight restorations and the cementation procedure were identical to those of procedure 1. After cementation, all restorations were finished and occlusion/articulation was checked and corrected when necessary.

### Placement Procedure 3: Direct Veneer Restorations

**Fig 8:**
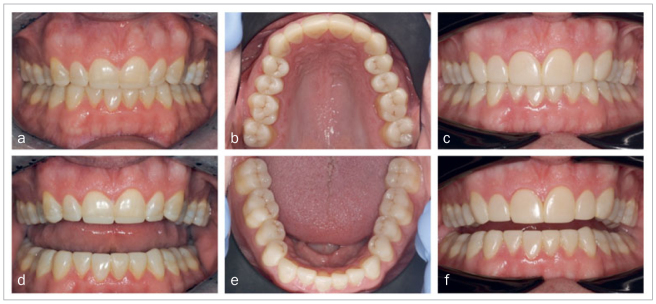
a) and d) before placement of buccal veneer restorations; b), c), e), f) after placement of buccal veneer restorations.

The placement of the CAD/CAM indirect restorations was followed by fabrication of direct composite buccal veneer restorations on the maxillary anterior teeth during placement procedure 3, one week after procedure 2. The indirect CAD/CAM palatal veneers were manufactured to their definitive length, which simplified direct veneer placement. After placement of a matrix, the buccal surface of the indirect restorations was roughened with a bur and air abraded with SiO_2_-coated Al_2_O_3_ (Cojet, 3M Oral Care). Then the tooth tissue was etched (Scotchbond Etchant, 3M Oral Care) for 15 s, rinsed, and dried. Silane was applied (ESPESIL, 3M Oral Care) and gently air dried, followed by adhesive application (Scotchbond Universal, 3M Oral Care), gentle air drying, and photocuring for 10 s. The composite resin (Filtek Supreme XTE, 3M Oral Care) was placed with a multi-layering technique, in this case using colors A2B and A1E. After light curing, the veneers were polished using fine diamond burs, Soflex discs (3M Oral Care), and rubber polishing disks (Twist DIA, Kuraray Noritake; Tokyo, Japan). Figure 8 shows the clinical result of this rehabilitation.

## Discussion

The aim of this case report was to describe the digital workflow used to restore a severely worn dentition with minimally invasive CAD/CAM resin nano-composite restorations. The treatment plan for this patient could have been counseling and monitoring to evaluate the progression of tooth wear. The patient, however, demanded treatment, and it was thus decided to start restorative treatment.^15^ Identification of etiological factors is important to appropriately assess risk factors. However, this is difficult and not always possible. Restorative treatment can be indicated in cases where tooth wear progresses rapidly or when severe clinical symptoms are present. There is no conclusive evidence that a specific treatment modality is more favorable in terms of long-term clinical performance for chemical or mechanical tooth wear. Here, it was decided to use a restorative treatment modality that combined CAD/CAM technology with minimally invasive dentistry.

The goal of treatment was to resolve functional problems and improve the quality of life of the patient by reconstructing the anatomical form of teeth through functional thickness of the restoration material. To restore lost tooth material, an increase of VDO was necessary, by which the temporo-mandibular joint opened. Because the joint opens by rotation first, the occlusal space for the posterior restorations was less than for the anterior restorations. Since anatomical form and VDO are not absolute, measurable clinical features, the new VDO in this procedure was established as a mixture of anticipated esthetics and optimal posterior occlusal space as judged by the dentist (Fig 3). To explore these features, we made a direct, free-hand mockup with composite in the mouth according to the lip-generated smile design,^20^ instead of the often-used provisional resin test build-up manufactured by the dental laboratory.^8,9,21,23,29^ The latter approach, when supported by a digital smile design technique,^4^ is a formal method to achieve personalized esthetics and can easily be transferred to the mouth by a mold. However, the digital 2D representation of esthetics in a 3D model is still complicated, while the composite mockup technique provides the patient a direct visualization of the new intraoral situation. However, making a direct mockup demands a high level of operator skill, as in a limited time frame, an acceptable and pleasing result for the patient must be obtained. Finally, the presence of challenges such as anterior open bite, edge-to-edge incisor relationship, or Angle Class II Division 1 occlusal relationship, influences the determination of a new VDO. In those cases, an increase in VDO complicates achieving an appropriate anterior occlusal relationship or tooth length. Under such circumstances, the treatment plan may require amendments, such as a smaller increase in VDO, or switching to a multidisciplinary approach, including orthodontic treatment planning, to achieve a predictable treatment result.

According to a systematic review,^1^ raising the VDO is considered a safe and predictable treatment for patients without TMD or dysfunctional habits. The review showed that the VDO can be increased up to 4 mm without causing problems. In the present case, the new VDO was applied without a test period, and has not resulted in any complaints or interventions/adjustments so far. The patient had adapted to the new VDO within a week. Some authors have included a period of adaptation to the increased VDO by wearing a removable device, eg, an acrylic splint.^9^ There is anecdotal data that the splint will hardly be worn during the day due to oral and social discomfort. This decreases its effectivity as a means of try-out. We therefore prefer to immediately increase VDO by fixed adhesive restorations, as described by others.^1,25,30^ It must be emphasized that a more gradual approach and use of a removable device is advised in patients who show signs of temporomandibular disorder.

In contradiction to functional dental rules, we did not provide the patient with a stable occlusion during the interim periods. The patient showed superior adaptability and overloading did not result in discomfort or pain. This type of trouble-free adaptation has also been reported in studies describing the Dahl method, which promotes eruption of non-worn teeth by applying supraoccluding restorations on the worn teeth.^2,6,12^ Complete rehabilitation of the dentition could be supported by the use of high-end occlusion and articulation registration instruments.^24^ As we have almost completely digital fabrication with precise digital occlusion registration, we consider scanning the maxillary and mandibular dentition in centric relation using bite blocks to be sufficient. Care was taken to have all restorations in static occlusal contact, including the anterior restorations. Active movements were guided by canine contacts to support posterior disclusion and by comfortable incisor contact. Moreover, bite registration was done in the desired new VDO and therefore no adjustments in VDO were necessary in the dental laboratory.

In these complex rehabilitations, uniform restorative materials on all teeth can be preferable, and we could have chosen to restore all teeth with direct resin composite restorations. It is reported that this type of direct treatment is quite common in treating severe tooth wear.^17,18^ However, building up posterior teeth with appropriate anatomical form and in adequate occlusal contact is strenuous and time-consuming. Indirect procedures, although costly, can be beneficial to obtain the required clinical quality in these cases. Although the CAD/CAM procedure used in this case study is simple, its treatment cost-effectiveness in comparison to that of direct restorations should be elucidated.

As the resin nano-ceramic material was only available in blocks of one color, the buccal veneers were made of directly applied composite veneers using multiple color shades. Using this type of resin material, we approached uniformity of restorative materials on all teeth. If the patient had had higher esthetic demands, individually adapted (resin) ceramic veneers in the anterior area could have been an alternative. Cases with occluding composite restorations in the posterior area and ceramic veneer restorations in the esthetic zone have been described earlier and offer a fine compromise between occlusal adaptability and stable esthetics.^31^

The reason the buccal and palatal/lingual veneers were placed in separate sessions had to do with time constraints: it was not possible to treat all anterior teeth in one session. A critical point of this two-step treatment procedure for anterior restorations is the bonding of the buccal veneer to the incisal edge of the palatal veneer. In severe wear patients who received direct composite veneers on both the palatal/lingual and the buccal surfaces of anterior teeth, it was shown that the incisal interface of the two veneers was at significant risk of debonding when these two restorations were placed in separate treatment sessions instead of in one treatment session.^16^ In this patient, we supported the adhesion by macro- and micromechanical roughening of the adhesive surfaces. In addition, silica coating with sandblasting and silanization were used prior to placement of the direct buccal veneer.

The veneer and table-top restorations have minimal retentive capacity, and intraoral occlusion check without the restorations being cemented was not possible. A positive finding was that the seating of the restorations was precise, and the occlusion did not require much finishing after placement. Since we had experienced that small discrepancies in the approximal areas of the restorations hampered the clinical seating of four tabletops in a row (eg, #34, 35, 36, and 37), we scanned and placed the posterior restorations in two separate procedures. Although this required at least one additional treatment session, the alternative – ie, adapting every single approximal surface before placing the restoration – would simply require even more time and effort.

## Conclusion

In the treatment of severe tooth wear, the described digital workflow using CAD/CAM resin nano ceramic for occluding restorations and direct composite materials in the esthetic zone is a potential treatment modality that is workable and minimally invasive. The free-hand mockup technique to explore esthetics and establish a new VDO was effective in this case.

## Clinical relevance:

This case report describes a digital workflow for CAD/CAM nanoceramic restoration of occlusal surfaces as a minimally invasive restorative treatment of severe tooth wear.
